# A pilot study on high amplitude low frequency–music impulse stimulation as an add‐on treatment for depression

**DOI:** 10.1002/brb3.1399

**Published:** 2019-09-11

**Authors:** Gudrun Agusta Sigurdardóttir, Peter Michael Nielsen, Jesper Rønager, August Gabriel Wang

**Affiliations:** ^1^ Psychiatric Centre Amager Copenhagen University Hospital Copenhagen S Denmark; ^2^ Department of Neurology Holbaek Hospital Sjaelland Denmark; ^3^ Department of Neurology Rigshospitalet, Copenhagen University Hospital Copenhagen Denmark

**Keywords:** depressive disorder/depression, pilot, randomized controlled trial, vagus nerve stimulation, vibroacoustic therapy

## Abstract

**Objective:**

High Amplitude Low Frequency–Music Impulse Stimulation (HALF‐MIS) is a form of Vagus Nerve Stimulation (VNS). The aim of the study was to determine the feasibility, efficacy, and potential side effects of HALF‐MIS, used as an add‐on treatment for depression.

**Methods:**

This is an open randomized controlled pilot study. Patients with depressive disorder were randomly allocated to either a HALF‐MIS group with eight add‐on HALF‐MIS sessions (over a period of 3–4 weeks) or a control group which received treatment as usual. Seated in a specially designed chair() embedded with a transducer, their central nervous system was stimulated through the abdomen, () using music and vibration. Hamilton rating was performed. Side effects were registered.

**Results:**

Eighteen patients were randomized to the add‐on treatment and 20 patients to the control group. Both groups show in Hamilton Depression Rating Scale (HDRS)‐17 and in HDRS‐6, although the HALF‐MIS group had a greater decline of symptoms. This was a significant difference in intergroup analysis (*p* = .011, CI 95% for the HALF‐MIS group 3.0588–8.5327 and CI 95% for the control group 0.2384–3.0). The (HDRS)‐6 difference was also significant (*p* = .020, CI 95% for the HALF‐MIS group 1.5911–5.0487 and for the control group −0.297 to 1.7058). No side effects were observed.

**Conclusions:**

High Amplitude Low Frequency–Music Impulse Stimulation treatment seems to give beneficial effect as an add‐on treatment for depression. HALF‐MIS appears to be a safe and effective add‐on treatment for depression.

## Graphical abstract

Music Impulse Stimulation (High Amplitude‐Low Frequency) is applied as an add‐on treatment for depression and shows promising results.

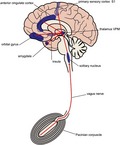

## SIGNIFICANT OUTCOMES AND LIMITATIONS

- The first study on HALF‐MIS as an add‐on treatment for depression.
- HALF‐MIS as an add‐on treatment for depression is more effective than standard treatment alone.
- Compared to treatment as usual, HDRS‐17, and HDRS‐6 showed significant reduction for the HALF‐MIS group.
- HALF‐MIS is a feasible and safe treatment without noticeable side effects.
- Open study.

## 1 INTRODUCTION

Depressive disorder is one of the most disabling illnesses worldwide. WHO has estimated that over 300 million people of all ages suffer from depression and that the burden of depression and other mental health conditions is on the rise globally (“WHO|Depression”, 2017). There is no cure for depression, and current treatments for major depression are only partially effective. Antidepressant response rates in controlled trials are estimated at ~54%, and real‐world effectiveness data suggest a somewhat lower rate (Murrough & Charney, 2012). Thus, there is much need for new treatments for depression.

The aim of this pilot study was to determine the feasibility, efficacy, and potential side effects of a new Danish treatment called High Amplitude Low Frequency – Music Impulse Stimulation (HALF‐MIS). HALF‐MIS was originally developed by a neurologist for treating pain and other neurological disorders. Anecdotal reports have, however, shown a good effect on patients' mood after receiving HALF‐MIS treatment. This led to curiosity about HALF‐MIS' effectiveness as an add‐on treatment for patients with clinical depressive disorder.

### 1.1 Vibroacoustic therapy

The HALF‐MIS modality resembles other vibroacoustic therapy (VAT) modalities, which was first described by the Norwegian therapist Olav Skille (Skille, 1989). Vibroacoustic therapy (VAT) uses sound to produce vibrations that are applied directly to the body. The vibrations are often utilized with music, where the vibration frequencies are provided as background to music which is composed to complement the frequencies. Music has been a medium of therapy for centuries, and there are numerous examples of the curative or healing powers of music in the historical records of different cultures (Maratos, Gold, Wang, & Crawford, 2008; Wigram, 1995). In HALF‐MIS and vibroacoustic therapy the key is the combined effect of music and vibrations.

Reports suggest that vibroacoustic therapy (VAT) can improve the treatment of stress induced depression, parkinsonism, Retts disease, autism, and other afflictions, including anxiety, tension, and fatigue (Skille, 1989). Wigram has written a review on the effect of vibroacoustic therapy (Wigram, 1995). Most previous studies are case histories, and the improvement in these cases(,) are often based on subjective assessments (Rüütel, 2002). There is a lack of randomized trials on the effect of VAT.

### 1.2 HALF‐MIS equipment and treatment session

**Figure 1:**
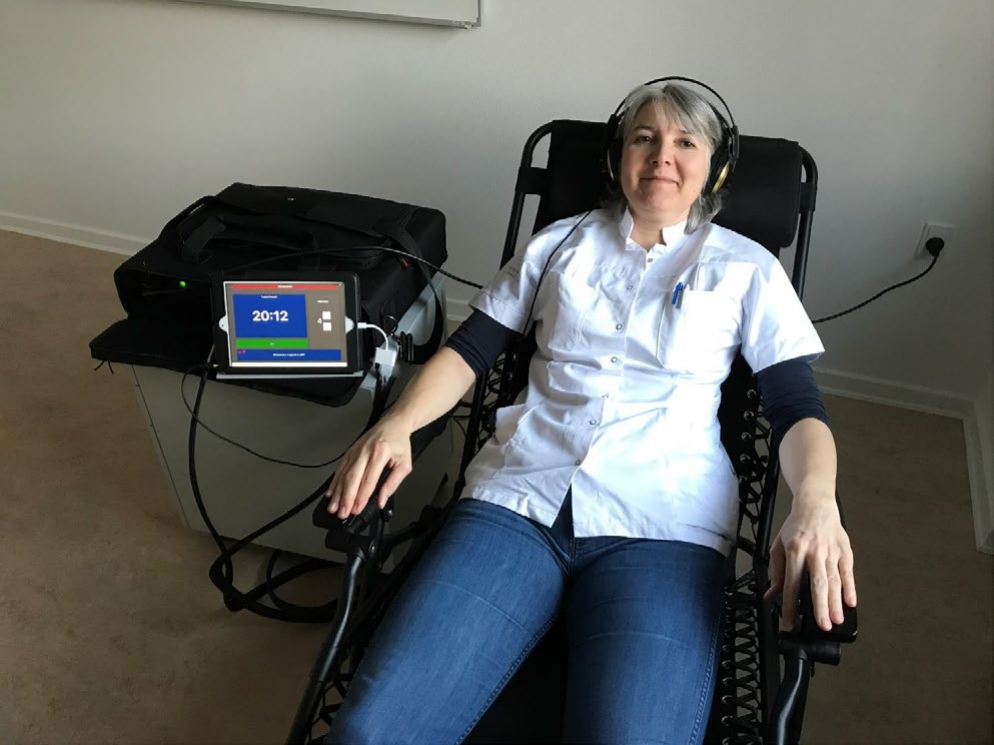
During a 20‐min HALF‐MIS session, the patient lies on a specially designed chair, embedded with a transducer, which transmits computer‐generated frequencies into vibrations. The vibrations are in sync with music the patient can hear through headphones. Thus, the central nervous system is stimulated through both the ears and the abdomen—using music and low‐frequency vibrations produced by the HALF‐MIS equipment

When a HALF‐MIS treatment session is initiated, the patient will put on headphones and take a seat in a zero‐gravity chair(,) with an electromechanical transducer unit fastened to the back of the chair (Figure 1). An iPad is connected to the central processing unit, comprising means for splitting and routing the signal and for amplification. The iPad has a user‐friendly HALF‐MIS app. When the app is open, the treatment screen emerges and the green “START” bar pops up. This bar is touched to start the session. The bar will turn red and flash a “STOP” sign. By touching the “STOP” sign(,) the patient will abort the session immediately. This is a safety precaution in case of malfunction or malaise. The HALF‐MIS music track and player software are installed in the app. The music and the software are specifically developed for the project, and the low‐frequency content is programmed in a separate track that is routed to the transducer. The music is composed, arranged, and produced so that the frequencies in this separate track are always synchronized to the audio track regarding rhythm and tuning.

### 1.3 HALF‐MIS effect on the body and brain

High Amplitude Low Frequency–Music Impulse Stimulation is a noninvasive, nonpharmaceutical, and nonelectric form of Vagus Nerve Stimulation (VNS). The Vagus nerve has afferents to the brain through the nucleus tractus solitarius, which further connects to the amygdala, the frontal cortex, and other brain areas that play important roles in cognitive emotion regulation and have been implicated in depression (Erk et al., 2010; Pandya, Altinay, Malone, Anand, 2012).

**Figure 2:**
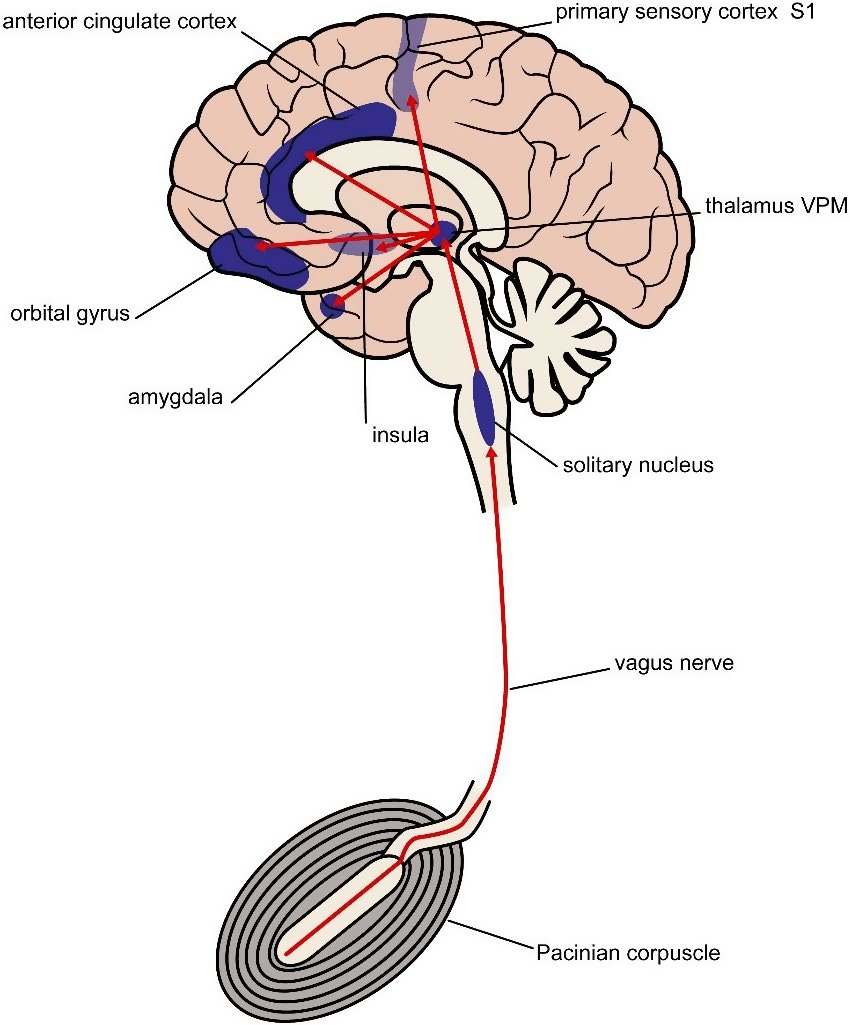
Vibrations from the HALF‐MIS modality activate Pacinian corpuscles, which send afferent impulses through the vagus nerve to cortical brain areas implicated in depressive disorder

Our hypothesis is that HALF‐MIS can stimulate the vagus nerve through the Pacinian corpuscles in the abdomen(,) in a way that affects central brain function (Figure 2) and thus has the potential to be beneficial in the treatment of depression. The mesentery and the inner organs have high concentrations of Pacinian corpuscles, up to 1 mm large vibration and pressure sensory organs. The Pacinian corpuscles send afferent impulses through the vagus nerve without synapsis until medulla oblongata, nucleus tractus solitarius as well as splanchnic nerves in well myelinated nerve fibers to the sensory ganglia of medulla spinalis. These impulses are distributed via thalamus(,)and the ventro‐postero‐lateral nuclei to the sensory cortex, the postcentral gyrus, SI and SII cortices, and to the limbic systems (Coghill et al., 1994). The cortical representation of the abdominal system of Pacinian corpuscles is unknown. There are few fast fibers in the abdominal part of the vagal nerve, but they may very well be Pacinian afferents. This has never been investigated. However, all the numerous vagal free nerve endings in the inner organs of the abdominal cave are stimulated too(,) when exposed to a powerful vibratory sound stimulus. Thus(,) it is inevitable that a vast flow of action potentials will propagate through all sensory afferents of the abdominal part of the vagal nerve.

The impulses from the Pacinian corpuscles are propagating through the nervous system at a maximal amplitude and speed. The function of the abdominal Pacinian corpuscles remains unclear. The free nerve endings of these Pacinian corpuscles are protected by layers of a keratin‐like substance arranged like coats in an onion. The Pacinian corpuscles have a maximal afferent output when stimulated with a frequency of 240 Hz (Bolanowski & Zwislocki, 1984), but no lower limit of frequency response has been found. Any stimulation will result in an afferent output. This means that the Pacinian corpuscles are activated by sound impulses even if these are imperceptible to the human ear (<20 Hz).

## 2 METHODS

The protocol was approved by the Danish Ethical Committee in February 2015. The study was performed at Psychiatric Centre Amager, Copenhagen University Hospital. Subjects included medically stable outpatients ages 18 through 70 years who met the ICD‐10 criteria for mild and moderate depression, lower Hamilton cut‐off at eight points. The diagnosis was verified by psychiatrists using ICD‐10 as well as Beck Depression Inventory (BDI). Before randomization, an experienced psychiatrist or psychologist performed a full Hamilton rating. Then randomization was performed, and the patients were thus included in either the HALF‐MIS group or the control group. Randomization method was a simple randomization, without use of strata or groups.

Exclusion criteria included pregnancy, earlier ECT treatment, psychotic features, or signs of serious suicidal or homicidal risk.

Patients in the HALF‐MIS group were scheduled for eight HALF‐MIS sessions over a period of 3–4 weeks. Each HALF‐MIS session lasted 20 min. The patients were asked to sit down comfortably in the chair and listen to the relaxing music. They were instructed how to stop the treatment and adjust the music volume (the vibration amplitude was fixed). The music was composed specifically for this purpose, and all patients listened to the same piece in all the sessions. The aim of the composition was to produce a calm and pleasant—yet not boring track. This was achieved by the use of predominant natural harmonics and avoiding dissonances and abrupt dynamics.

Clinicians continuously tracked the patients for adverse events and side effects, including asking for symptoms from all major organs and functions. At the end of the patients' eighth and last session, Hamilton rating was performed again. For the control group, who had treatment as usual, the last Hamilton rating was performed 3–4 weeks after beginning standard treatment and joining the study. The patients from the control group were also offered the HALF‐MIS treatment after finishing the control period. This was for ethical reasons only, and after serving as controls they were not included in the HALF‐MIS group.

Characteristics for the two groups were compared: Age, gender, and time from Hamilton start to finish. Comparison of age and time was done by *t* test. Comparison of gender contribution was tested by Chi‐square test. The results from the Hamilton ratings (both from 17‐items scale and 6‐items scale) were calculated by the general linear model. All statistics by SPSS 25.

All subjects received treatment as usual. During the project most of the patients were treated either with Serotonin Selective Reuptake Inhibitors (SSRI) or Serotonin–Norepinephrine Reuptake Inhibitors (SNRI) antidepressants (10/18 of the HALF‐MIS group and 16/20 of the controls).Two patients from the HALF‐MIS group and one from the control group were treated with Noradrenergic, a specific serotonergic antidepressants (NaSSA). Five patients from the HALF‐MIS group and two from the control group were in transition from one type antidepressant to another.

## 3 RESULTS

Thirty‐eight patients participated. By randomization 18 patients were in the treatment group (TAU and HALF‐MIS) versus 20 patients in the control group (TAU and also opportunity to be on a waiting list for HALF‐MIS after 3–4 weeks). Only one patient left the study, due to assignment to ECT treatment. At the start all patients were in depression treatment. Some characteristics recorded are presented in Table 1. The two groups were compared according to age, gender, and time (time from Hamilton rating at start to Hamilton rating at end).

**Table 1:** Some characteristics of the two groups

	Gender		Age	Time from start to finish	Patients in psychotherapy
	Male	Female	Average years	Days	
HALF‐MIS	8	10	37.7	26.2	9
Control	5	15	41.1	24.6	11

Abbreviations: HALF‐MIS, High Amplitude Low Frequency–Music Impulse Stimulation.

Age was comparable in the two groups, *p* = .445 (*t* test). There were more women in the control group, although not significantly across groups, *p* = .207. Proportions in psychotherapy were comparable for the two groups, *p* = .757. Treatment periods were comparable for the two groups, *p* = .141 (*t* test).

Table 2 shows results for both Hamilton rating scales with 17 items (HDRS‐17) and six items (HDRS‐6). For the HALF‐MIS group, the difference for HDRS‐17 from start to end was 5.55 (highly significant with *p* = .001) and for the control group the difference was 1.60 (also significant with *p* = .019). The difference in HDRS‐6 for the HALF‐MIS group was 3.12 (*p* = .004) while the difference for the control group was 0.75 (*p* = .121). Thus both groups show a reduction in HDRS‐17, although the HALF‐MIS group had a greater symptom reduction. As to the HDRS‐6, the reduction is more evident for the HALF‐MIS group. The HDRS‐17 difference was significant in intergroup analysis (*p* = .011). For the HALF‐MIS group CI 95% (3.059–8.533) and for the control group CI 95% (0.238–3.050). The (HDRS)‐6 difference was also significant (*p* = .020). For the HALF‐MIS group CI 95% (1.591–5.049) and for the control group CI 95% (−0.2970 to 1.706). 50% reduction or more in HDRS‐17 is a definition of a responder. 4/18 in the HALF‐MIS group versus 0/20 in the control group were responders, a significant difference (Chi‐square test, *p* = .026, Fishers Exact test, *p* = .041). A seven points reduction is a definition of a remitter. 5/18 versus 1/20 were remitters; this is only a marginally significant difference (Chi‐square test, *p* = .055; Fishers Exact test, *p* = .083).

**Table 2:** Results and statistics from HDRS‐17 and HDRS‐6

	HDRS‐17 start	HDRS‐17 end	Intergroup HDRS‐17 *p*‐value	HDRS‐6 start	HDRS‐6 end	Intergroup HDRS‐6 *p*‐value
HALF‐MIS	20.77	15.22	.011	11.11	7.88	.020
Control	19.65	18.05		10.35	9.60	

Abbreviations: HALF‐MIS, High Amplitude Low Frequency–Music Impulse Stimulation; HDRS, Hamilton Depression Rating Scale.

Concerning side effects, two HALF‐MIS patients reported some unexpected effects. One patient experienced an outburst of crying at the start of the two first sessions. Another patient experienced a chilly feeling at the first session only. In both cases, no somatic explanation was found.

## 4 DISCUSSION

The primary results show a significant improvement in depressive ratings when comparing HALF‐MIS as an add‐on treatment to treatment as usual alone. Even with this small sample size (*n* = 38) and open registry, these promising findings show that the HALF‐MIS treatment is feasible, effective, safe, well tolerated, and worthy of further study. A limitation is that the study protocol was not published before the study, neither on http://www.clinicaltrials.gov nor any similar website.

Interestingly, there was significant reduction in the 6‐items Hamilton rating scale (HDRS‐6) for the HALF‐MIS group, from 11.11 to 7.88 (*p* = .004) whereas the control group had a reduction from 10.35 to 9.60 (*p* = .121). The HDRS‐6 scale has been found strongly correlated to other versions of the HDRS both at baseline and after treatment, but also as sensitive to change of treatment (O'Sullivan, Fava, Agustin, Baer, & Rosenbaum, 1997). HDRS‐6 measures the core depression features. This core depression may be characterized by feelings of sadness, hopelessness and guilt, a decrease in interest or time spent on activities, decreased motor activity and ability to concentrate, a lack of energy, and an increase in tension, irritability, and worry (Hooper & Bakish, 2000).

It would be interesting to see if HALF‐MIS alone can improve mood or somatic symptoms in general. There are, however, many elements of the treatment that isolated could explain the HALF‐MIS effect, such as the vibration alone, the music therapy, a 20‐min meditation session in a comfortable chair or simply doctor's effect. The placebo effect is shown to be substantial in depression treatment, maybe equal to antidepressant medication (Reazza et al., 2018). Therefore, blinded randomized controlled trials are needed.

The unsatisfactory results associated with conventional treatments for depression demonstrate a need for research into additional therapies. Vagus Nerve Stimulation (VNS) has been shown to be efficacious for the long‐term management of patients with treatment‐resistant depression (Carreno & Frazer, 2017; Johnson & Wilson, 2018; Rush et al., 2000). A recent 5‐year observational study of patients with treatment‐resistant depression treated with VNS or treatment as usual has provided additional evidence that adjunctive VNS has enhanced antidepressant effects compared with treatment as usual (Aaronson et al., 2017; Rush et al., 2005). However, the traditional VNS is invasive with a pacemaker‐like device which is implanted in the chest and neck during general anesthesia. Given the risks and inconvenience of surgical procedures, one could argue that non‐invasive VNS would be preferable for psychiatric patients who often suffer from other chronic physical conditions or multimorbidity (Read, Sharpe, Modini, & Dear, 2017). HALF‐MIS is not only a non‐invasive type of VNS, but also non‐electric compared to ECT, and non‐chemical. A portable device such as HALF‐MIS that can deliver stimulation to the vagus nerve through the abdomen looks promising for psychiatry if proven effective in further studies.

## CONFLICT OF INTEREST

Peter Michael Nielsen (PMN) and Jesper Ronager (JR) are registered investor/co‐inventor of the HALF‐MIS technology described in the patent WO 2015/144185 A1. Although there has been no product developed and no commercialization of the IP yet, this may be considered a conflict of interest. As a consequence, PMN and JR have not participated in either patient information/inclusion, rating of symptoms, data handling/statistic processing, or even in the discussion of the results. Otherwise no conflict of interest.

## Data Availability

The data that support the findings of this study are available from the corresponding author upon reasonable request.
